# The long-term effect of biologics in patients with ulcerative colitis emerging from a large Japanese cohort

**DOI:** 10.1038/s41598-022-25218-x

**Published:** 2022-12-06

**Authors:** Yuya Yokoyama, Yuki Ohta, Sadahisa Ogasawara, Jun Kato, Ryoko Arai, Hirotaka Koseki, Masaya Saito, Tatsuya Kaneko, Mamoru Tokunaga, Hirotaka Oura, Tsubasa Oike, Yushi Imai, Kengo Kanayama, Naoki Akizue, Junichiro Kumagai, Takashi Taida, Kenichiro Okimoto, Keiko Saito, Yoshihiko Ooka, Tomoaki Matsumura, Tomoo Nakagawa, Makoto Arai, Tatsuro Katsuno, Yoshihiro Fukuda, Yoshio Kitsukawa, Naoya Kato

**Affiliations:** 1 Department of Gastroenterology, Graduate School of Medicine, Chiba University, 1-8-1 Inohana, Chuo-Ku, Chiba, 260-8670 Japan; 2 Translational Research and Development Center, Chiba University Hospital, Chiba, Japan; 3 Department of Gastroenterology, Chiba Aoba Municipal Hospital, Chiba, Japan; 4 Department of Gastroenterology, Seikeikai Chiba Medical Center, Chiba, Japan; 5 Department of Medical Oncology, Graduate School of Medicine, Chiba University, Chiba, Japan

**Keywords:** Gastrointestinal diseases, Gastrointestinal system, Gastrointestinal diseases, Immunological disorders

## Abstract

To gain a better understanding of the effects of biologics, we evaluated clinical outcomes in patients with moderate to severe exacerbations of ulcerative colitis (UC). This retrospective, multicenter study retrieved the entire clinical courses of UC patients who began treatments between 2004 and 2018. All exacerbations and clinical parameters, including treatment details for exacerbations and both remission and re-exacerbation dates, were identified during the observation period. Two different endpoints, the cumulative incidence rates of surgical resection and re-exacerbation, were evaluated separately in moderate to severe exacerbation events. Among 1401 patients, 1626 exacerbation events were determined according to a partial Mayo score (remission: < 2, mild: 2–4, moderate: 5–7, and severe: > 7). During the observation period, as administration rates of biologics increased, both surgical resection and hospitalization rates decreased, for 959 moderate to severe exacerbation events. We confirmed that biologics significantly reduced the cumulative re-exacerbation rate in moderate to severe exacerbation events during the study period compared with suboptimal therapies (a 0.507-fold decreased risk according to COX regression analysis, *P* < 0.001). However, they had not enough impact in reducing the cumulative incidence rate of surgical resection in moderate to severe exacerbation events that were corticosteroid-refractory or dependent (a 0.878-fold decreased risk according to COX regression analysis, *P* = 0.606). Biologics may improve remission duration, but these agents had no significant impact in reducing the risk of surgical resection in moderate to severe active UC.

## Introduction

Ulcerative colitis (UC), a subtype of inflammatory bowel disease (IBD), is a chronic inflammatory disease of the colon, representing the most common distressing period of productive age and resulting in disability^1^. Although UC is associated with the Caucasian population in the United States and Europe, its incidence in newly developed countries, primarily in Asia, has been increasing rapidly over the past few decades^2–5^. Japan ranks second in the world in terms of the number of UC patients after the United States^3–6^. The sharp increase in UC cases in Japan appears to be associated with Westernized diets and environments, which affect the intestinal microbiome and increase the risk for UC in genetically susceptible individuals^7,8^.

The primary treatment goals for UC patients are to induce and maintain remission. Specifically, surgical resection of the total colon due to an induction failure and an early re-exacerbation after remission are the two worst clinical outcomes in patients with UC^1^. 5-aminosalicylates (5-ASA), which are anti-inflammatory agents, are the major choice of treatment to induce and maintain remission in patients with mild to moderate active UC^9,10^. Corticosteroids are a classical and most widely used treatment for inducing remission based on their confirmed high rates of immediate effectiveness^11–13^. Over half a century since the first report, corticosteroids remain the first-line treatment for moderate to severe exacerbations or when 5-ASA proves ineffective, as recommended by global guidelines ^14–17^. However, roughly one-third of patients have not achieved clinical response or remission, while almost two-thirds of patients had required reintroduction of corticosteroids within two years and even they had achieved remission^18,19^.

During the past two decades, there has been a dramatic paradigm shift in UC treatment with the introduction of biologics, primarily antitumor necrosis factor (anti-TNF) therapy, for both inducing and maintaining remission^9,14,15,17^. Well-designed randomized controlled trials have confirmed that biologics, including infliximab, adalimumab, golimumab, and vedolizumab, both improved induction and sustained remission rates compared with placebo in patients with moderate to severe active UC who had failed corticosteroid therapy or who had a history of corticosteroid refractory or dependent^20–24^. The rates of surgical resection and hospitalization in UC patients have decreased in both Eastern and Western populations^25,26^. However, the effects of biologics for patients with active UC, particularly focusing on overall long-term effectiveness of these new agents for maintaining remission, remain controversial and have not yet been clarified sufficiently. Therefore, this study was conducted to evaluate the clinical outcomes of biologics in UC patients with moderate to severe exacerbations using a large retrospective Japanese cohort over a long observation period.

## Methods

### Study population and study design

Chiba City is inhabited by approximately 1 million people (the 12th largest population in Japan) with less migration compared with the central metropolitan area of Japan. Chiba University Hospital, Chiba Aoba Municipal Hospital, and Chiba Medical Center are the only three institutions to which well-trained specialists in IBD belong.

We retrospectively investigated the entire clinical courses of UC patients by reviewing the electronic medical records (EMR) in three institutions and identified all exacerbations according to the partial Mayo score^27^. We identified all exacerbation events during the entire observation period and acquired the following data: the date of exacerbation diagnosis, laboratory data, and disease extent at the time of exacerbation, requirement of hospitalization, and medical treatments for exacerbation.

All participating hospitals received approval from IRB. The Institutional Review Board of Chiba University Hospital approved this study (approval number: 3399).

Informed consent was waived because this was a survey study using medical records and no written or verbal consent could be obtained from the research subjects. However, materials regarding opting out were posted to give patients the opportunity to refuse to participate in the study. We conduct our research in accordance with the "Ethical Guidelines for Medical Research Involving Human Subjects” established by the Ministry of Education, Culture, Sports, Science and Technology and the Ministry of Health, Labour and Welfare of Japan.

### Treatment strategies for UC

UC diagnosis was based on the combination of conventional clinical, endoscopic, radiological, and pathological criteria^28^. 5-ASA is the first choice for inducing and maintaining remission in patients with mild to moderate active UC. Corticosteroids are introduced for patients with moderate to severe active UC, and/or in those unresponsive to 5-ASA, and are tapered off over 8–12 weeks. Biologics (infliximab (IFX), adalimumab (ADA) and golimumab (GLM)), and immunomodulators (IM) (i.e., thiopurines; azathioprine/6-mercaptopurine), were considered in case of corticosteroid failure. Calcineurin inhibitors (CNIs), apheresis, and surgical resection were considered rescue medical treatments in patients who were unresponsive to corticosteroids. Since tofacitinib and vedolizumab were approved in Japan in 2018 and had been used by only a few patients, we excluded these patients from this study.

### Definition of exacerbations according to partial Mayo score

The degree of exacerbations during the observation period was assessed according to the partial Mayo score using three noninvasive parameters (stool frequency, rectum bleeding, and physician’s global assessment)^26^. Each of the three categories was rated from 0 to 3 which was summed up to derive a total score in the ranges of 0–9 (remission: < 2, mild: 2–4, moderate: 5–7, and severe: > 7). Moderate and severe exacerbations were defined as partial Mayo scores of 5–7 and > 7, respectively. The remission date was defined as the first date after the exacerbation event that we confirmed as clinical improvement according to a partial Mayo < 2. The re-exacerbation date was defined as the first date after remission that we confirmed a change in clinical status according to a partial Mayo score ≥ 2.

### Assessments of clinical outcomes of corticosteroids

Clinical outcomes of corticosteroids were evaluated for each exacerbation event. Corticosteroid-refractory was defined if remission, which was assessed according to a partial Mayo score < 2, could not be achieved by corticosteroid monotherapy. Meanwhile, cases of active disease revival while receiving reduced doses of corticosteroids were regarded as corticosteroid-dependent. We collectively reviewed the cases that showed corticosteroid-refractory or -dependent (COR-REF or DEP) in the analysis.

### Definition of effectiveness evaluation and COR-REF or DEP cohorts

All moderate to severe active events in UC patients were classified as an effectiveness evaluation cohort for assessing the clinical outcomes of treatments during induction of, and maintaining remission after, excluding events that were inducted by 5-ASA alone. COR-REF or DEP cohorts were defined when any of the following conditions were observed: (1) met the abovementioned criteria of COR-REF or DEP after taking corticosteroids for moderate to severe exacerbations or (2) had a previous history of the criteria of COR-REF or DEP inducted for exacerbations using treatments without corticosteroids.

### Statistical analysis

Pearson’s chi-squared test or Fisher’s exact test was used, as appropriate. ANOVA test was used to compare the mean between independent groups. The cumulative incidence of surgical resection was analyzed using Kaplan–Meier plots, which was defined as the duration from the date of exacerbation till the date of surgical resection, with the censoring date defined as the day of the last follow-up or the date of re-exacerbation. The cumulative re-exacerbation incidence was analyzed using Kaplan–Meier plots, which was defined as the duration from the date of exacerbation to the date of re-exacerbation, with the censoring date defined as the day of the last follow-up. Cox regression analyses were performed to evaluate the factors for the cumulative risk of surgical resection and re-exacerbation in UC patients with moderate to severe exacerbations. All statistical analyses were conducted using the SPSS statistical software (version 25; SPSS-IBM, Chicago, IL, USA).

## Results

### Study population and baseline characteristics

Between January 2004 and December 2018, 1401 patients were started on treatments for UC at the three institutions and were included in the present retrospective cohort. The final data were locked on December 2019, and the median observation period was 48.3 months (95% CI: 44.7–51.9); the median number of relapses was 0, and 451 of the 1401 patients had relapses. Supplementary Fig. 1 depicts the study population trend per year. The number of follow-up patients per year increased dramatically, and hospitalization rates per year decreased in the present cohort.

Table 1 shows the baseline characteristic of the study population at the time of the initial visit during the study period. The mean age was 39.8 years, and one-fourth of the patients were aged ≤ 22.0 years. Supplementary Fig. 2 presents the correlations between the year of onset and the mean age at initial UC diagnosis. The mean age at initial diagnosis appeared to be significantly higher along with the calendar year of onset (P < 0.001).

**Table 1:** Baseline characteristics at the initial visit.

Demographics/characteristics	Any (n = 1401)
Gender, male (*n* [%])	762 (54.4)
**Age at the time of the first consultation, years**	
Mean (SD)	39.8 (17.3)
Median (range)	38.0 (0–93)
Percentile (25%)	26
Percentile (50%)	38
Percentile (75%)	52
**Age at the time of the initial diagnosis, years**	
Mean (SD)	35.0 (16.7)
Median (range)	31.5 (0–93)
Percentile (25%)	22
Percentile (50%)	31.5
Percentile (75%)	46
Height, cm, median (SD)	162.9 (9.7)
Body weight, kg, median (SD)	57.0 (12.0)
Body mass index, median (SD)	19.0 (7.6)
**Smoking**	
None (*n* [%])	676 (48.3)
Previous smoker (*n* [%])	186 (13.3)
Current smoker (*n* [%])	80 (5.7)
Unknown (*n* [%])	459 (32.8)
Prior history of appendectomy (*n* [%])	24 (1.7)
Initial diagnosis of UC at the time of first consultation (*n* [%])	419 (29.9)
**Partial Mayo grade at the time of the first consultation**	
Inactive (*n* [%])	475 (33.9)
Mild (*n* [%])	451 (32.2)
Moderate (*n* [%])	329 (23.5)
Severe (*n* [%])	146 (10.4)
**Type of UC at the time of the first consultation**	
Proctitis (*n* [%])	367 (26.2)
Left-sided colitis (*n* [%])	368 (26.3)
Pancolitis (*n* [%])	488 (34.8)
Right-sided or segmental colitis (*n* [%])	95 (6.8)
Unknown (*n* [%])	83 (5.9)

### Clinical characteristics, treatments, and clinical outcomes for moderate to severe exacerbations

**Figure 1:**
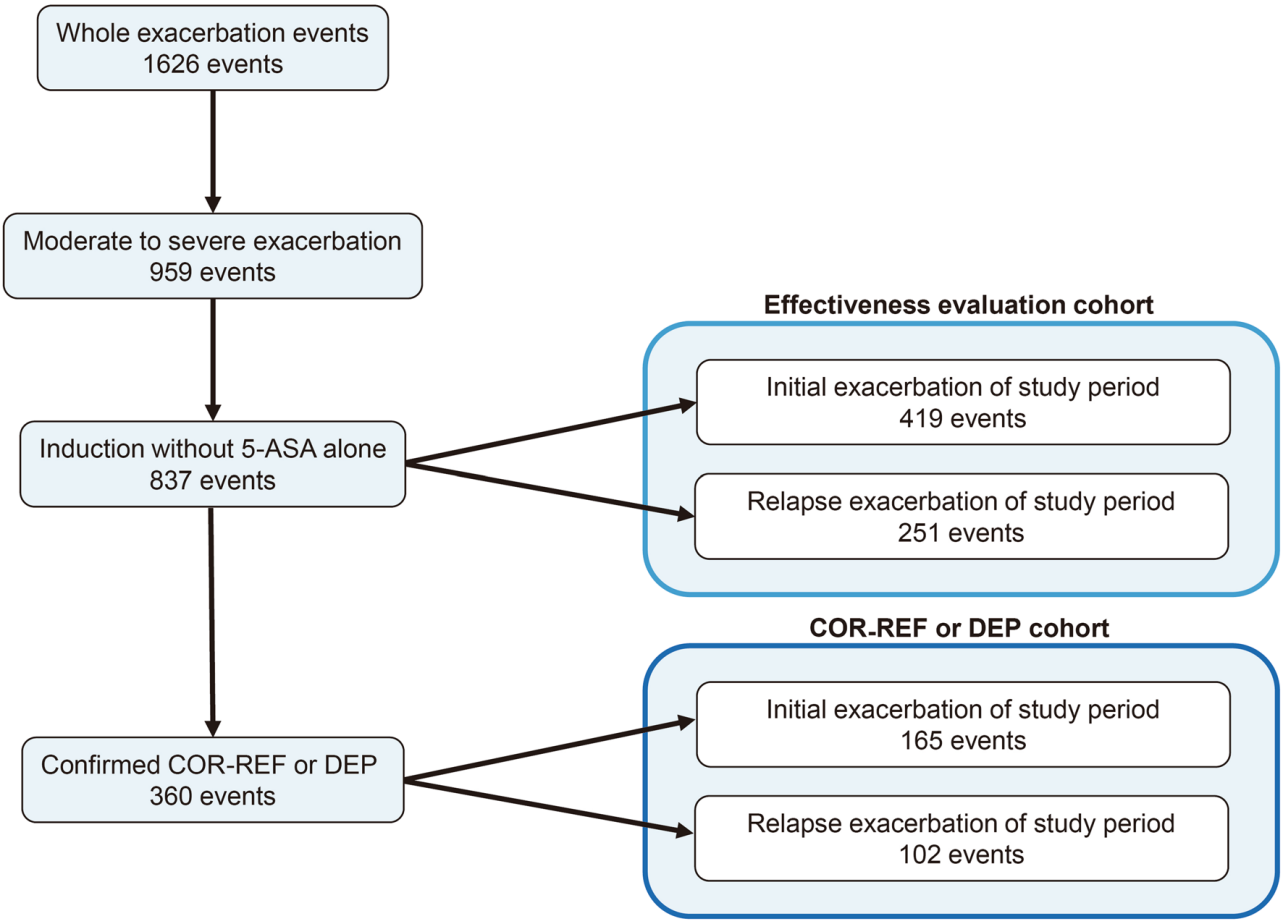
Study flow.

We identified 1639 exacerbation events from the study cohort. Then, 13 exacerbation events were excluded from this analysis due to using vedolizumab and tofacitinib for the treatment. Of 1626 exacerbation events to be analyzed, 959 were classified as moderate to severe as follows: 207 in 2004–2008 (21.6%), 304 in 2009–2013 (31.7%), and 448 in 2014–2018 (46.7%) (Fig. 1, upper left column). Of the moderate to severe exacerbation events among the entire study population, 878 (91.5%) were confirmed as remission; 61 (6.4%) patients in the cohort never achieved remission, among whom 41 (4.3%) underwent surgery.

Next, we identified 837 events from the overall exacerbations that were moderate to severe exacerbations and treated with 5-ASA alone or more. We also extracted 360 events classified as COR-REF or DEP.

We also extracted the first and second exacerbations of each patient’s clinical course during the observation period and identified them as an effectiveness evaluation cohort, COR-REF cohort, or DEP cohort (Fig. 1, right column).

From now on, we will refer to the first exacerbation of study period as initial exacerbation, and the second exacerbation of study period as relapse exacerbation (see Supplementary Fig. 4). Table 2 shows the clinical characteristics of the overall, initial, and relapse exacerbations in patients with moderate to severe active UC.

**Table 2:** Clinical characteristics and treatments in patients with moderate to severe UC exacerbations.

Characteristics	Overall exacerbations	Initial exacerbations	Relapse exacerbations
	(n = 959)	(n = 525)	(n = 266)
Age, year, mean (SD)	38.6 (17.4)	38.1 (17.9)	38.3 (16.9)
Partial Mayo, severe (n [%])	246 (25.6)	155 (29.5)	59 (22.2)
Pancolitis UC (n [%])	403 (42.0)	210 (40.0)	115 (43.2)
**Laboratory data, mean (SD)**			
White blood cell	8924.8 (3769.8)	9201.2 (3891.9)	8736.7 (3637.8)
Serum albumin	3.76 (0.71)	3.61 (0.79)	3.99 (0.532)
C-reactive protein	2.70 (4.36)	3.23 (4.83)	2.06 (3.42)
Hospitalization (n [%])	372 (38.8)	236 (45.0)	85 (31.7)
**Treatments for exacerbations (n [%])**			
5-ASAs	330 (34.4)	292 (55.6)	29 (10.9)
Single-use	124 (12.9)	106 (20.2)	15 (5.6)
Corticosteroids	628 (65.5)	342 (65.1)	179 (67.2)
Immunomodulators	78 (8.1)	39 (7.4)	20 (7.5)
Calcineurin inhibitors	204 (21.3)	110 (21.0)	61 (22.9)
Biologics	164 (17.1)	55 (10.5)	41 (15.4)
Apheresis	254 (26.5)	133 (25.3)	78 (29.3)
Surgical resection	81 (8.4)	42 (8.0)	22 (8.3)

**Figure 2:**
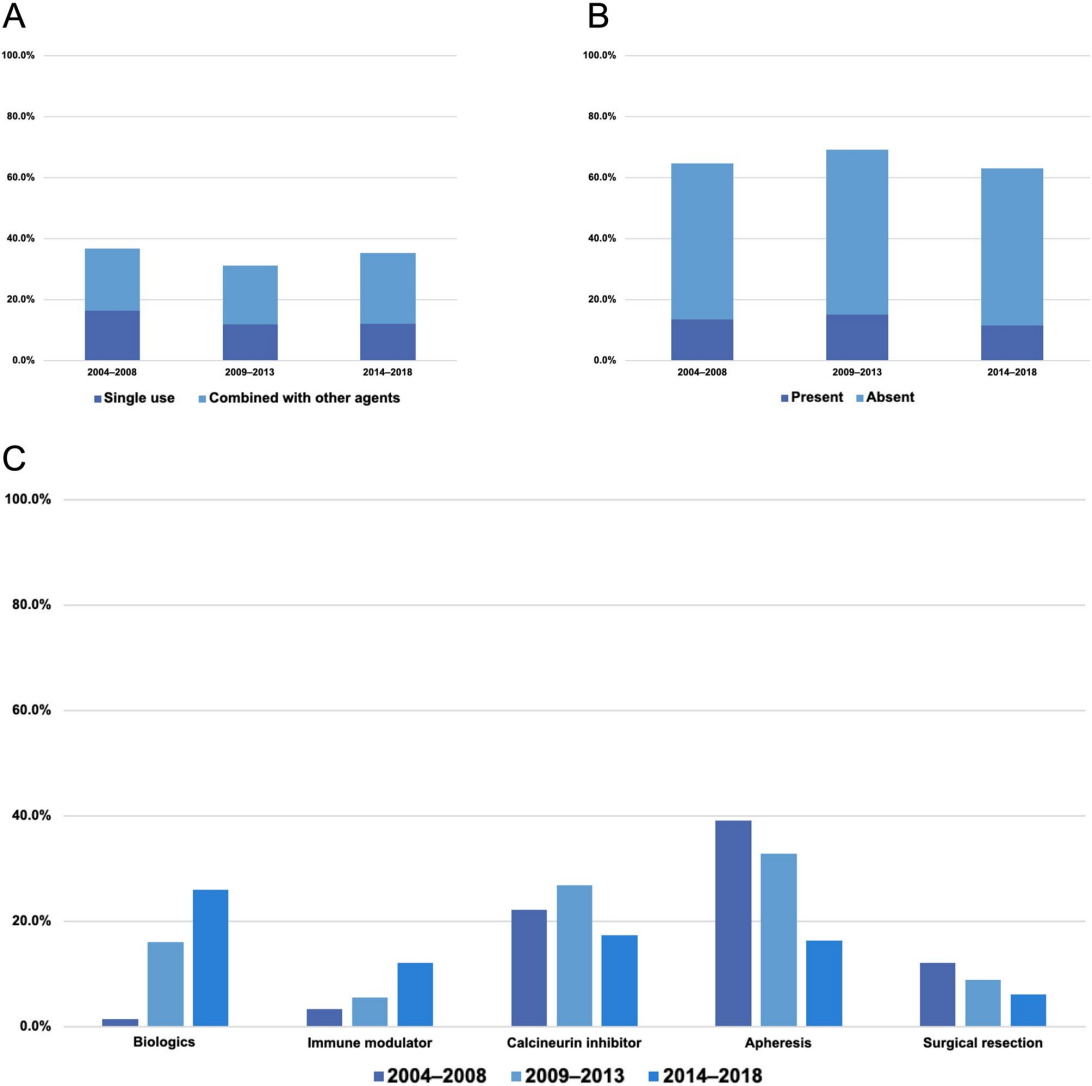
Transitions of treatments in patients with moderate to severe ulcerative colitis exacerbations. (**A**) 5-Aminosalicylates, (**B**) Corticosteroids (“present” means previous history of COR-REF or DEP), (**C**) Other treatments.

5-ASA was administered in 330 of 959 moderate to severe exacerbation events (34.4%), of which 122 (12.7%) were treated with 5-ASA alone. Single-use rates of 5-ASA for moderate to severe exacerbations were similar in the three time periods (Fig. 2). We excluded those events that were introduced by 5-ASA alone (n = 122) and set an effectiveness evaluation cohort in the present study (Fig. 1, n = 837). Median time periods to remission and re-exacerbation of overall moderate to severe exacerbations in the effectiveness evaluation cohort were 1.7 months (95% CI, 1.6–18.9) and 36.4 months (95% CI, 29.5–43.3), respectively. In addition, the medication status in this cohort differed before and after the introduction of molecular-targeted drugs, which is shown in Supplementary Fig. 3.

Corticosteroids were used for 628 events (65.5%) of moderate to severe active UC, of which 126 (13.0% of overall moderate to severe exacerbations and 20.0% of inductions by corticosteroids) had a previous history of corticosteroid refractory. The proportions of the previous history of COR-REF or DEP were almost in concordance with the three time periods (Fig. 2, *P* = 0.581). During the study period, 56.4% of events were confirmed as remission by corticosteroids. The remission rates of corticosteroids were significantly higher if there was no previous history of COR-REF or DEP (no COR-REF or DEP: 60.6%, COR-REF or DEP: 39.7%, *P* < 0.001). Of 837 events in the effectiveness evaluation cohort, we classified 360 events as COR-REF or DEP cohort, based on our earlier definition (Fig. 1, the right-lower column). In the COR-REF or DEP cohort, IFX, ADA, and GLM were administrated to 79 (62%), 53 (41%), and 26 (20%) patients, respectively.

Figure 2C depicts the transitions of treatments for moderate to severe exacerbations. Biologics showed an increasing tendency during the study period. In contrast, surgical resection and apheresis showed a gradually decreasing trend during the same period.

### Cumulative risk of surgical resection in UC patients with moderate to severe exacerbations

Based on the effectiveness evaluation cohort of overall exacerbation events, we analyzed factors for the cumulative risk of surgical resection in patients with moderate to severe active UC. A multivariate Cox regression model revealed age ≥ 60 years, severe exacerbations, history of COR-REF or DEP, induction by apheresis, and calcineurin inhibitors were independent risk factors for surgical resection (Table 3). On the other hand, steroids tended to reduce the risk of surgery, although the multivariate analysis did not show a significant difference. Biologics had no significant effect on the cumulative risk of surgical resection.

**Table 3:** Cox regression analysis of the factors for cumulative risk of surgical resection in patients with moderate to severe UC exacerbations.

Variables	Univariate analysis		*P*	Multivariate analysis		*P*
	Hazard ratio	95% CI		Hazard ratio	95% CI	
Age, ≥ 60 years	1.895	1.133–3.170	0.015	2.269	1.341–3.840	0.002
First attack	0.825	0.436–1.560	0.554	0.997	0.511–1.945	0.993
Pancolitis UC	1.432	0.924–2.221	0.108	1.156	0.737–1.813	0.528
Severe exacerbation	3.954	2.545–6.142	< 0.001	2.973	1.860–4.754	< 0.001
History of COR-REF or DEP	3.986	2.576–6.167	< 0.001	2.920	1.806–4.720	< 0.001
Corticosteroids	0.569	0.368–0.879	0.011	0.666	0.419–1.057	0.085
Apheresis	2.021	1.302–3.136	0.002	1.807	1.132–2.884	0.013
Calcineurin inhibitors	5.059	3.246–7.884	< 0.001	2.695	1.657–4.383	< 0.001
Biologics	1.355	0.849–2.164	0.203	0.913	0.556–1.499	0.719

**Figure 3:**
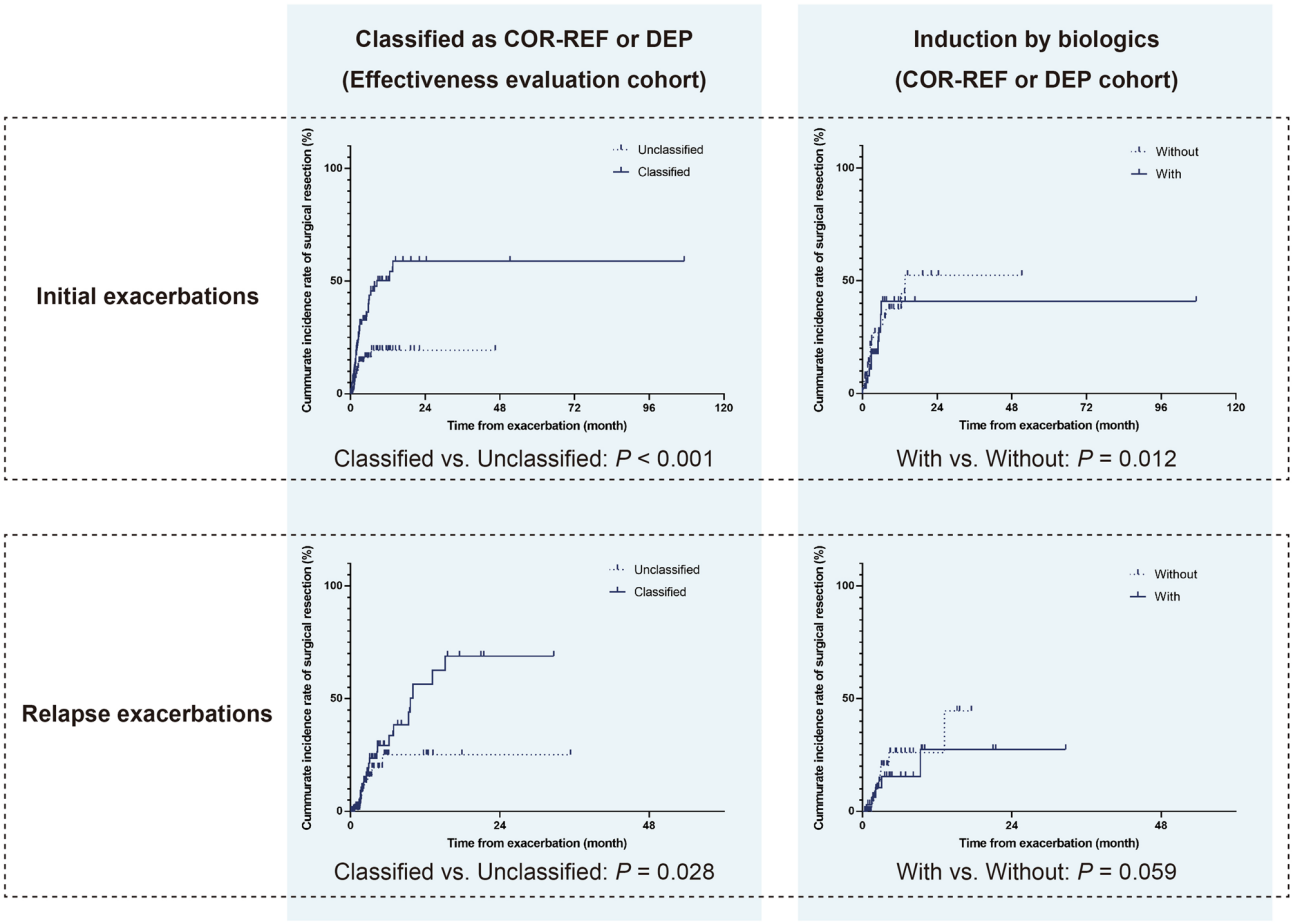
Cumulative incidences of surgical resection in patients with moderate to severe ulcerative colitis exacerbations. The left column: the impact of classified as COR-REF or DEP cohort on cumulative incidence of surgical resection in patients with the effective evaluation cohort. The right column: impact of induction by biologics on cumulative incidence of surgical resection in patients with the COR-REF or DEP cohort.

We next verified the cumulative incidence of surgical resection in the effectiveness evaluation cohorts of initial, and relapse exacerbation events in subgroups (Fig. 3). Classified as COR-REF or DEP were significantly high risk for surgical resection in moderate to severe exacerbation events (Fig. 3, the left column). However, biologics did not have a significant effect in reducing the risk for surgical resection (Fig. 3, the right column).

### Cumulative risk of re-exacerbation in UC patients with moderate to severe exacerbations

**Figure 4:**
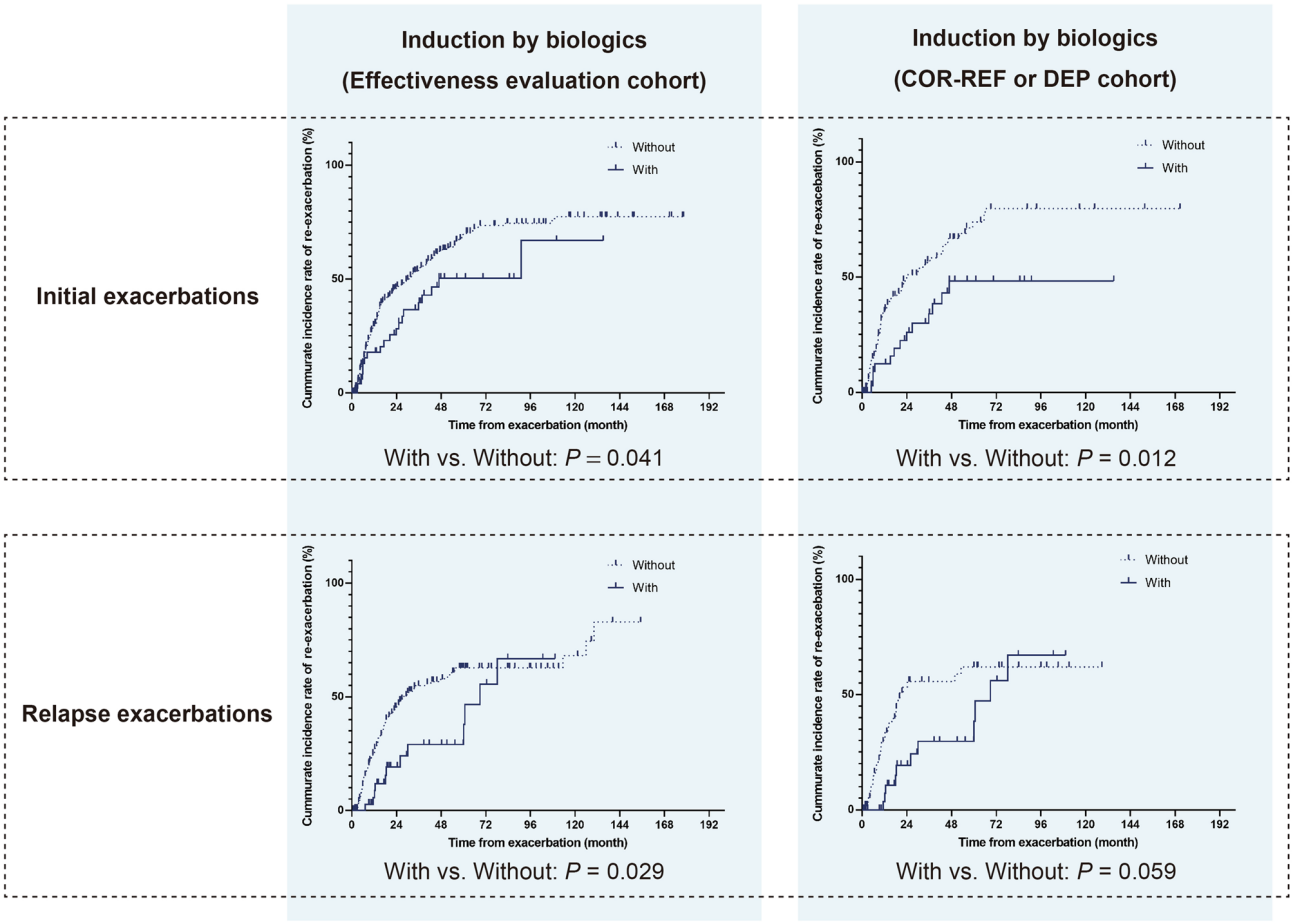
Cumulative incidences of re-exacerbations in patients with moderate to severe ulcerative colitis exacerbations. The left column: the impact of induction of biologics on the cumulative incidence of re-exacerbation in patients with the effectiveness evaluation cohort. The right column: the impact of induction of biologics on the cumulative incidence of re-exacerbation in patients with the COR-REF or DEP cohort.

Results of the multivariate Cox regression model in overall moderate to severe exacerbation events in patients with UC are shown in Table 4. Biologics significantly reduced the risk of re-exacerbation. In contrast, induction by corticosteroids significantly increased the risk of re-exacerbation. Figure 4 shows the Kaplan–Meier curve of the cumulative incidence of re-exacerbation caused due to the administration of biologics. Our results confirmed that biologics reduced the risk of re-exacerbation in the initial, and relapse exacerbations population in the effective evaluation cohort (Fig. 4, the left column). We also evaluated the impact of induction of biologics on the cumulative incidence rate of re-exacerbation in patients with COR-REF or DEP cohort. The cumulative incidence of re-exacerbations in the initial exacerbation group decreased, but the decrease in the cumulative incidence of re-exacerbations in the relapse exacerbation group was not significant. (Fig. 4, the right column).

**Table 4:** Cox regression analysis of the factors for cumulative risk of re-exacerbation in patients with moderate to severe UC exacerbations.

Variables	Univariate analysis		*P*	Multivariate analysis		*P*
	Hazard ratio	95% CI		Hazard ratio	95% CI	
Age, ≥ 60 years	0.828	0.611–1.115	0.214	0.813	0.603–1.096	0.174
First attack	0.976	0.771–1.236	0.839	0.803	0.612–1.054	0.113
Pancolitis UC	1.227	1.015–1.484	0.035	1.217	1.065–1.391	0.004
Severe exacerbation	1.238	1.000–1.533	0.050	1.133	0.902–1.424	0.283
History of COR-REF or DEP	0.846	0.664–1.077	0.173	0.930	0.716–1.209	0.589
5-ASA	1.046	0.859–1.274	0.652	1.161	0.911–1.480	0.226
Corticosteroids	1.436	1.164–1.772	0.001	1.473	1.183–1.833	0.001
Apheresis	0.992	0.803–1.226	0.944	0.977	0.785–1.216	0.837
Calcineurin inhibitors	1.115	0.886–1.404	0.353	1.206	0.939–1.549	0.142
Immunomodulators	1.051	0.754–1.467	0.768	1.066	0.760–1.480	0.712
Biologics	0.527	0.389–0.713	< 0.001	0.501	0.367–0.683	< 0.001

## Discussion

We demonstrated the effect of biologics in patients with moderate to severe active UC in the Japanese real-world practice by setting two different endpoints, i.e., the cumulative incidence rate of surgical resection and re-exacerbation. Our findings indicated the distinctive potential of biologics and the strategic directions of using these drugs in patients.

This study established a unique endpoint, i.e., the cumulative incidence rate of re-exacerbation, and focused on the clinical impact of biologics on maintaining remission in moderate to severe exacerbation events of UC. In general, the clinical outcomes of biologics were assessed using the remission rate. Most randomized controlled trials of biologics have generally compared with placebo by selecting 44-, 52-, or 54-week remission rate as the primary endpoint^20–24^. Recent studies that confirmed the effectiveness of biologics used similar indicators^29,30^. Even though remission rate is a defined and convenient endpoint to assess the effectiveness of both induction and maintenance of remission in UC patients, endpoints with 44-, 52-, or 54-week remission rate appear to be suitable for clinical trials because it does not require long observation periods. However, considering the disease characteristics of UC, the potential of treatments should be evaluated separately based on induction and maintenance of remission. We believe that our study is the first one to demonstrate the cumulative incidence rate of re-exacerbation of biologics in moderate to severe active UC using cohorts from real-world practice. Our results suggested that the administration of biologics significantly reduced the risk of re-exacerbation and prolonged remission duration in moderate to severe UC exacerbation.

In this study, 63%, 28%, and 28% of the Biologics used in all events were IFX, ADA, and GLM, respectively, which are all anti-TNFα antibody drugs.

Since IFX was used in many cases and previous reports have shown no significant difference in the efficacy of maintenance treatment with anti-TNFα antibody drugs, we reviewed them together as biologics^31^.

The cumulative risk of re-exacerbation events, which achieved remission by corticosteroids, was significantly higher than that achieved using biologics in the COR-REF or DEP cohort of our study. Altogether, these results demonstrated an important finding that the conspicuous effect of biologics is maintaining remission in patients with active UC. Based on the results of the present study, biologics may be used not only in COR-REF or DEP patients, but also in patients achieving remission by corticosteroids for maintaining remission. We suggest that subpopulation of patients achieving remission by corticosteroids who have a high risk of surgical resection in case of re-exacerbation have indications for biologics. Although the medical cost of biologics is a huge medical problem^32,33^, biosimilar and generic drugs may help to solve the issue^34^.

There are some limitations in this study. First, this was a retrospective study, but we believe that this design was necessary to conduct a comparative study before and after the introduction of biologics. Second, this research was conducted within a limited area in Japan. Therefore, bias may exist when compared with a nationwide study. However, most of the IBD cases in a city of 1 million people are covered by the medical institutions in this cohort, and we consider this to be practical data.

Finally, this study did not include newer molecular target drugs, such as Tofacitinib, Vedolizumab, and Ustekinumab. Future studies should include these agents.

Our study also confirmed the potential of corticosteroids in reducing the risk of surgical resection in moderate to severe UC exacerbations, although statistical significance was not observed with the multivariate analysis. Corticosteroids’ potential for achieving remission and avoiding surgical resection should be re-recognized in moderate to severe active UC as reported previously^11–13,18,19^. Conversely, biologics showed no dramatic impact on reducing surgical resection risk compared with suboptimal treatments in the COR-REF or DEP population. In this cohort, 28 of the surgical cases received biologic agents, of which 13 (46%) were severe cases and one (3%) developed cancer; furthermore, the median time from deterioration to surgery was 83 days.　Although a randomized controlled trial verified that infliximab reduced the risk of surgical resection compared with placebo^20^, a recent study reported by Murthy et al. demonstrated that anti-TNF therapy did not decrease UC-related intestinal resections in real-world practice^35^. Altogether, these results and ours indicate that biologics might not have the anticipated effectiveness for avoiding resection, especially in salvaging the COR-REF or DEP population with rapid exacerbation. Hence, there is a strong need to develop promising agents with similar or higher potential of achieving remission compared to corticosteroids.

The characteristics of the present retrospective cohort were generally illustrative to represent the Japanese UC population during the past few decades. First, both the number of UC patients and the median age at onset had been increasing^36–38^. Second, the rates of hospitalization and surgical resection in the entire population had decreased gradually, including other countries as well^26,39^. Surgical resection rates of the present cohort were similar those of a Korean cohort^26^, although these rates were lower than those of a Western cohort^40^. Remarkably, the proportion of using immunomodulators (i.e., thiopurines) in the present cohort was lower than that in Western countries^41^. It is well known that the incidence rates of adverse events, primarily leukopenia and hair loss, are higher in East Asian populations, including Japan, than in Caucasian populations^42,43^. Recently, Moriyama et al. reported that a variant in the nudix hydrolase 15 (NUDT15) gene (R139C, c415C > T) was associated with early severe leukopenia in Asians^44^. Currently, the assessment of NUDT R139C has been approved by the Japanese regulatory authority. Consequently, the opportunity to use thiopurines should increase. It would be necessary to confirm the clinical impact of thiopurines in Japanese UC patients in the near future. Regarding biologics, the administration rate is increasing, as in the rest of the world, and is almost equal or slightly higher than those in other countries due to the well-supported system of medical costs^26,35^. Biologics will become more essential agents for treating active UC patients, due to active development of biologics in the coming years^45,46^. We anticipate further studies in the future to investigate the effectiveness of biologics focusing on maintaining remission in other cohorts from all over the world.

## Conclusion

Our study has confirmed that biologics significantly improved the duration of maintaining remission compared with suboptimal treatments in patients with moderate to severe active UC in a large retrospective Japanese cohort. However, they could not exert a significant effect in reducing the risk of surgical resection in patients with moderate to severe active UC with corticosteroid refractory. We believe that our study results would help us gain a deeper understanding of the characteristics of biologics used for treating UC patients.

## Supplementary Information

Supplementary Information.

## Data Availability

The datasets used and/or analyzed during the current study are available from the corresponding author on reasonable request. IRB did not permit data sharing, because we did not inform patients of data sharing.

## References

[1] Ungaro R, Mehandru S, Allen PB, Peyrin-Biroulet L, Colombel JF (2017). Ulcerative colitis. Lancet.

[2] Molodecky NA (2012). Increasing incidence and prevalence of the inflammatory bowel diseases with time, based on systematic review. Gastroenterology.

[3] Ng SC (2017). Worldwide incidence and prevalence of inflammatory bowel disease in the 21st century: A systematic review of population-based studies. Lancet.

[4] Thia KT, Loftus EV, Sandborn WJ, Yang SK (2008). An update on the epidemiology of inflammatory bowel disease in Asia. Am. J. Gastroenterol..

[5] Yang Y, Owyang C, Wu GD (2016). East Meets West: The increasing incidence of inflammatory bowel disease in Asia as a paradigm for environmental effects on the pathogenesis of immune-mediated disease. Gastroenterology.

[6] Murakami Y (2019). Estimated prevalence of ulcerative colitis and Crohn's disease in Japan in 2014: An analysis of a nationwide survey. J. Gastroenterol..

[7] Kaplan GG, Ng SC (2017). Understanding and preventing the global increase of inflammatory bowel disease. Gastroenterology.

[8] Ng SC (2015). Environmental risk factors in inflammatory bowel disease: A population-based case-control study in Asia-Pacific. Gut.

[9] Bressler B (2015). Clinical practice guidelines for the medical management of nonhospitalized ulcerative colitis: The Toronto consensus. Gastroenterology.

[10] Ford AC (2011). Efficacy of 5-aminosalicylates in ulcerative colitis: systematic review and meta-analysis. Am. J. Gastroenterol..

[11] Truelove SC, Witts LJ (1955). Cortisone in ulcerative colitis; final report on a therapeutic trial. Br. Med. J..

[12] Ford AC (2011). Glucocorticosteroid therapy in inflammatory bowel disease: systematic review and meta-analysis. Am. J. Gastroenterol..

[13] Garcia-Planella E (2012). Long-term outcome of ulcerative colitis in patients who achieve clinical remission with a first course of corticosteroids. Dig. Liver Dis..

[14] Feuerstein JD (2020). AGA clinical practice guidelines on the management of moderate to severe ulcerative colitis. Gastroenterology.

[15] Matsuoka K (2018). Evidence-based clinical practice guidelines for inflammatory bowel disease. J. Gastroenterol..

[16] Ooi CJ (2010). The Asia-Pacific consensus on ulcerative colitis. J Gastroenterol Hepatol.

[17] Harbord M (2017). Third European evidence-based consensus on diagnosis and management of ulcerative colitis. Part 2: Current management. J. Crohns Colitis.

[18] Järnerot G, Rolny P, Sandberg-Gertzén H (1985). Intensive intravenous treatment of ulcerative colitis. Gastroenterology.

[19] D'Haens G (2001). Intravenous cyclosporine versus intravenous corticosteroids as single therapy for severe attacks of ulcerative colitis. Gastroenterology.

[20] Rutgeerts P (2005). Infliximab for induction and maintenance therapy for ulcerative colitis. N. Engl. J. Med..

[21] Reinisch W (2011). Adalimumab for induction of clinical remission in moderately to severely active ulcerative colitis: Results of a randomised controlled trial. Gut.

[22] Sandborn WJ (2012). Adalimumab induces and maintains clinical remission in patients with moderate-to-severe ulcerative colitis. Gastroenterology.

[23] Cunningham G, Samaan MA, Irving PM (2019). Golimumab in the treatment of ulcerative colitis. Ther. Adv. Gastroenterol..

[24] Feagan BG (2013). Vedolizumab as induction and maintenance therapy for ulcerative colitis. N. Engl. J. Med..

[25] Parragi L (2018). Colectomy rates in ulcerative colitis are low and decreasing: 10-year follow-up data from the swiss IBD cohort study. J. Crohns Colitis.

[26] Cha JM (2020). Long-term prognosis of ulcerative colitis and its temporal changes between 1986 and 2015 in a population-based cohort in the Songpa-Kangdong district of Seoul, Korea. Gut.

[27] Lewis JD (2008). Use of the noninvasive components of the Mayo score to assess clinical response in ulcerative colitis. Inflamm. Bowel Dis..

[28] Loftus EV (2000). Ulcerative colitis in Olmsted County, Minnesota, 1940–1993: Incidence, prevalence, and survival. Gut.

[29] Sands BE (2019). Vedolizumab versus adalimumab for moderate-to-severe ulcerative colitis. N. Engl. J. Med..

[30] Narula N (2018). Vedolizumab for Ulcerative Colitis: Treatment Outcomes from the VICTORY Consortium. Am J Gastroenterol.

[31] Kawalec P, Pilc A (2016). An indirect comparison of infliximab versus adalimumab or golimumab for active ulcerative colitis. Arch. Med. Sci..

[32] Wu B, Wang Z, Zhang Q (2018). Cost-effectiveness of different strategies for the treatment of moderate-to-severe ulcerative colitis. Inflamm. Bowel Dis..

[33] Stawowczyk E, Kawalec P (2018). A systematic review of the cost-effectiveness of biologics for ulcerative colitis. Pharmacoeconomics.

[34] Meyer A (2019). The effectiveness and safety of infliximab compared with biosimilar CT-P13, in 3112 patients with ulcerative colitis. Aliment. Pharmacol. Ther..

[35] Murthy SK (2020). Introduction of anti-TNF therapy has not yielded expected declines in hospitalisation and intestinal resection rates in inflammatory bowel diseases: A population-based interrupted time series study. Gut.

[36] Komoto S (2018). Clinical differences between elderly-onset ulcerative colitis and non-elderly-onset ulcerative colitis: A nationwide survey data in Japan. J. Gastroenterol. Hepatol..

[37] Higashiyama M (2019). Management of elderly ulcerative colitis in Japan. J. Gastroenterol..

[38] Fujimoto T (2007). Change of clinical characteristics of ulcerative colitis in Japan: Analysis of 844 hospital-based patients from 1981 to 2000. Eur. J. Gastroenterol. Hepatol..

[39] Fumery M (2018). Natural history of adult ulcerative colitis in population-based cohorts: A systematic review. Clin. Gastroenterol. Hepatol..

[40] Frolkis AD (2013). Risk of surgery for inflammatory bowel diseases has decreased over time: A systematic review and meta-analysis of population-based studies. Gastroenterology.

[41] Eriksson C (2017). Changes in medical management and colectomy rates: a population-based cohort study on the epidemiology and natural history of ulcerative colitis in Örebro, Sweden, 1963–2010. Aliment. Pharmacol. Ther..

[42] Hibi T, Naganuma M, Kitahora T, Kinjyo F, Shimoyama T (2003). Low-dose azathioprine is effective and safe for maintenance of remission in patients with ulcerative colitis. J. Gastroenterol..

[43] Takatsu N (2009). Adverse reactions to azathioprine cannot be predicted by thiopurine S-methyltransferase genotype in Japanese patients with inflammatory bowel disease. J. Gastroenterol. Hepatol..

[44] Moriyama T (2016). NUDT15 polymorphisms alter thiopurine metabolism and hematopoietic toxicity. Nat. Genet..

[45] Sabino J, Verstockt B, Vermeire S, Ferrante M (2019). New biologics and small molecules in inflammatory bowel disease: An update. Ther. Adv. Gastroenterol..

[46] Danese S (2020). New drugs in the ulcerative colitis pipeline: Prometheus unbound. Gastroenterology.

