# Supplementary material for: The long-term effect of biologics in patients with ulcerative colitis emerging from a large Japanese cohort
Source: Sci Rep. 2022 Dec 6;12:21060. doi: 10.1038/s41598-022-25218-x (PMC9727107; doi:10.1038/s41598-022-25218-x)

**The long-term effect of biologics in patients with ulcerative colitis emerging from a large Japanese cohort**

Yuya Yokoyama¶[1], Yuki Ohta¶[1], Sadahisa Ogasawara¶[1,2], Jun Kato*[1], Ryoko Arai[1], Hirotaka Koseki[3], Masaya Saito[4], Tatsuya Kaneko[1], Mamoru Tokunaga[1], Hirotaka Oura[1], Tsubasa Oike[1], Yushi Imai[1], Kengo Kanayama[1], Naoki Akizue[1], Junichiro Kumagai[1], Takashi Taida[1], Kenichiro Okimoto[1], Keiko Saito[1], Yoshihiko Ooka[1], Tomoaki Matsumura[1], Tomoo Nakagawa[1], Makoto Arai[1,5], Tatsuro Katsuno[1], Yoshihiro Fukuda[4], Yoshio Kitsukawa[3], Naoya Kato[1]

[1]Department of Gastroenterology, Graduate School of Medicine, Chiba University, Chiba, Japan

[2]Translational Research and Development Center, Chiba University Hospital, Chiba, Japan

[3]Department of Gastroenterology, Chiba Aoba Municipal Hospital, Chiba, Japan

[4]Department of Gastroenterology, Seikeikai Chiba medical center, Chiba, Japan

[5]Department of Medical Oncology, Graduate School of Medicine, Chiba University, Chiba, Japan

**Supplementary Figure 1. Transitions of followed up number of patients (A), proportions of worst exacerbations (B), and hospitalization rates (C) per year in patients with ulcerative colitis in the present cohort.**

**Supplementary Figure 2. Transitions of correlations between age of onset and years in patients with ulcerative colitis in Japan.**

**Supplementary Figure 3. Transition of treatments used before and in the bio-era.**

**Supplementary Figure 4. Definition of initial and relapse exacerbations in this cohort.**

Suppl. Figure 1 Yokoyama et al.

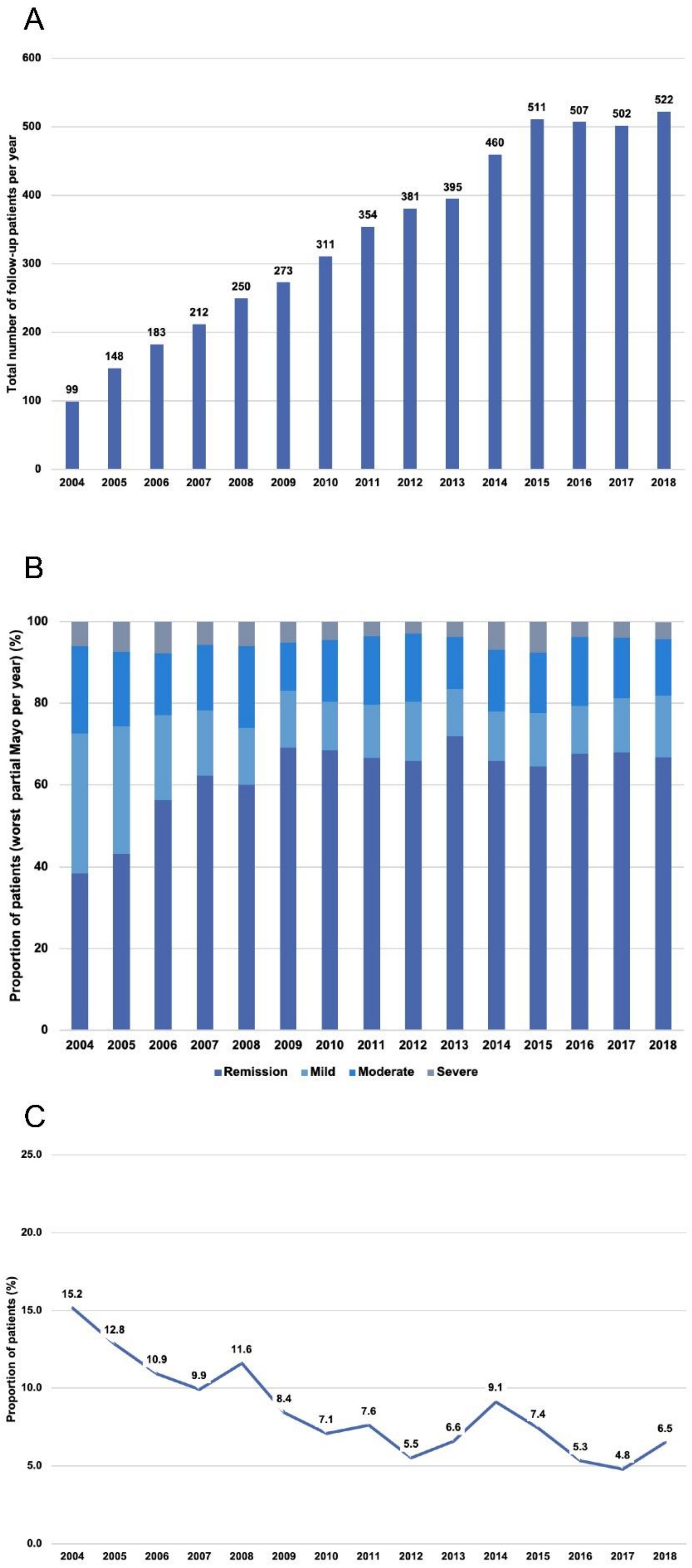

Suppl. Figure 2 Yokoyama et al.

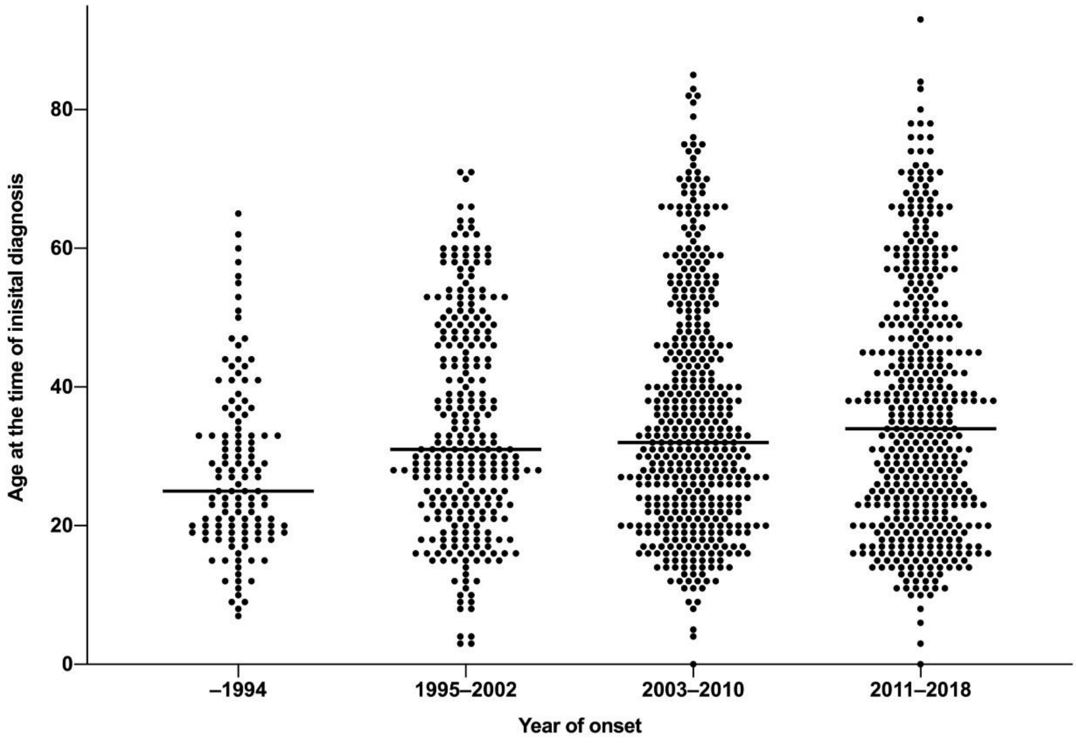


Suppl. Figure 3 Yokoyama et al.

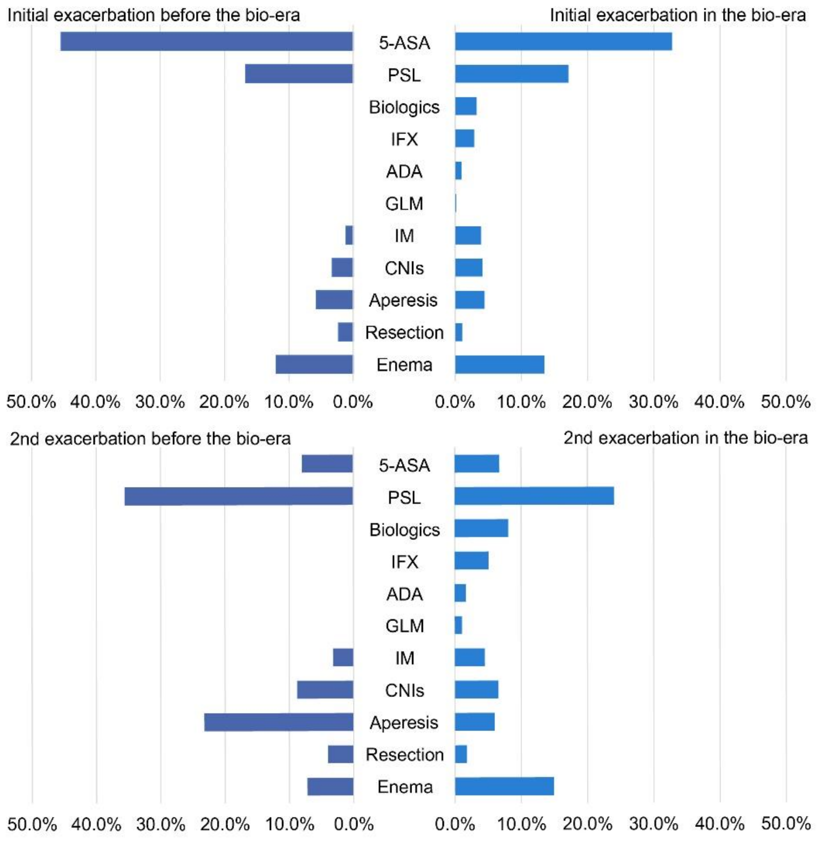

Suppl. Figure 4 Yokoyama et al.

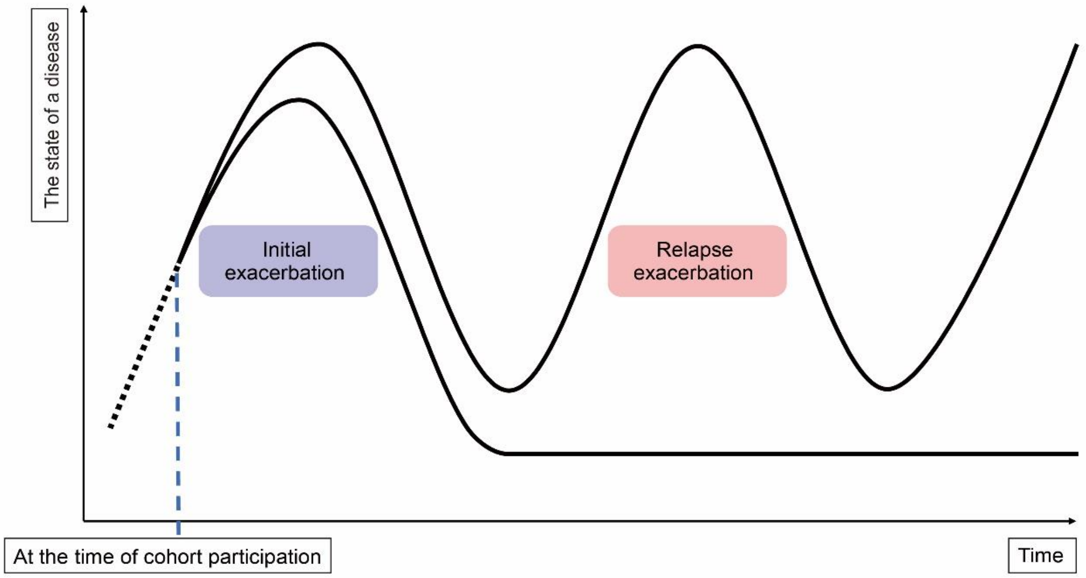

Supplement: Supplementary file 1 — Supplementary Information. [file 41598_2022_25218_MOESM1_ESM.pdf]
